# Supplementary material for: Multigene phylogeny and taxonomy of Dendryphion hydei and Torula hydei spp. nov. from herbaceous litter in northern Thailand
Source: PLoS One. 2020 Feb 5;15(2):e0228067. doi: 10.1371/journal.pone.0228067 (PMC7001993; doi:10.1371/journal.pone.0228067)

PLOS ONE

Multigene phylogeny and taxonomy of Torula hydei and Dendryphion hydei spp. nov. from herbaceous litter in northern Thailand

--Manuscript Draft--

**Manuscript Number:**	PONE-D-19-27091
**Article Type:**	Research Article
**Full Title:**	Multigene phylogeny and taxonomy of Torula hydei and Dendryphion hydei spp. nov. from herbaceous litter in northern Thailand
**Short Title:**	Torula hydei and Dendryphion hydei spp. nov. from herbaceous litter in northern Thailand
**Corresponding Author:**	ITTHAYAKORN PROMPUTTHA, Ph.D.<br>Chiang Mai University Faculty of Science<br>Muang Chiang Mai, Chiang Mai THAILAND
**Keywords:**	2 new species; Dothideomycetes; Hyphomycetes; Pleosporales; Torulaceae
**Abstract:**	Asexual fungi are some of the most significant microorganisms involved in decomposition of plants and contribute to nutrient recycling. During our studies on asexual fungi colonizing herbaceous litter in northern Thailand, we discovered two new fungal species, viz. Torula hydei and Dendryphion hydei spp. nov. The latter are examined, and their morphological characters are described as well as their DNA sequences from ribosomal and protein coding genes are analysed to infer their phylogenetic relationships with extant fungi. Torula hydei is different from other similar Torula species in having tiny and catenate conidia. Dendryphion hydei can be distinguished from other similar Dendryphion species in having large conidiophores and subhyaline to pale olivaceous brown, 2–4(–5)-septate conidia. Multigene phylogenetic analyses of a combined LSU, SSU, TEF1-α, RPB2 and ITS DNA sequence dataset generated from maximum likelihood and Bayesian inference analyses indicate that T. hydei forms a distinct lineage and basal to T. fici. Dendryphion hydei forms a distinct lineage and basal to D. europaeum, D. comosum, D. aquaticum and D. fluminicola within Torulaceae (Pleosporales, Dothideomycetes).
**Order of Authors:**	Junfu Li
	Rajesh Jeewon
	Peter E. Mortimer
	Mingkwan Doilom
	Rungtiwa Phookamsak
	ITTHAYAKORN PROMPUTTHA, Ph.D.
**Additional Information:**	
**Question**	**Response**
**Financial Disclosure**<br><br>Enter a financial disclosure statement that describes the sources of funding for the work included in this submission. Review the submission guidelines for detailed requirements. View published research articles from *PLOS ONE* for specific examples.<br><br>This statement is required for submission and **will appear in the published article** if	No

Powered by Editorial Manager® and ProduXion Manager® from Aries Systems Corporation

the submission is accepted. Please make sure it is accurate.<br><br>**Unfunded studies**<br>Enter: *The author(s) received no specific funding for this work.*<br><br>**Funded studies**<br>Enter a statement with the following details:<br>• Initials of the authors who received each award<br>• Grant numbers awarded to each author<br>• The full name of each funder<br>• URL of each funder website<br>• Did the sponsors or funders play any role in the study design, data collection and analysis, decision to publish, or preparation of the manuscript?<br>• **NO** - Include this sentence at the end of your statement: *The funders had no role in study design, data collection and analysis, decision to publish, or preparation of the manuscript.*<br>• **YES** - Specify the role(s) played.<br><br>* typeset	
**Competing Interests**<br><br>Use the instructions below to enter a competing interest statement for this submission. On behalf of all authors, disclose any competing interests that could be perceived to bias this work—acknowledging all financial support and any other relevant financial or non-financial competing interests.<br><br>This statement **will appear in the published article** if the submission is accepted. Please make sure it is accurate. View published research articles from *PLOS ONE* for specific examples.	NO authors have competing interests

Powered by Editorial Manager® and ProduXion Manager® from Aries Systems Corporation

**NO authors have competing interests**<br><br>Enter: *The authors have declared that no competing interests exist.*<br><br>**Authors with competing interests**<br><br>Enter competing interest details beginning with this statement:<br><br>*I have read the journal's policy and the authors of this manuscript have the following competing interests: [insert competing interests here]*<br><br>* typeset	
**Ethics Statement**<br><br>Enter an ethics statement for this submission. This statement is required if the study involved:<br><br>• Human participants<br>• Human specimens or tissue<br>• Vertebrate animals or cephalopods<br>• Vertebrate embryos or tissues<br>• Field research<br><br>Write "N/A" if the submission does not require an ethics statement.<br><br>General guidance is provided below. Consult the submission guidelines for detailed instructions. **Make sure that all information entered here is included in the Methods section of the manuscript.**	N/A

Powered by Editorial Manager® and ProduXion Manager® from Aries Systems Corporation

**Format for specific study types**<br><br>**Human Subject Research (involving human participants and/or tissue)**<br>• Give the name of the institutional review board or ethics committee that approved the study<br>• Include the approval number and/or a statement indicating approval of this research<br>• Indicate the form of consent obtained (written/oral) or the reason that consent was not obtained (e.g. the data were analyzed anonymously)<br><br>**Animal Research (involving vertebrate animals, embryos or tissues)**<br>• Provide the name of the Institutional Animal Care and Use Committee (IACUC) or other relevant ethics board that reviewed the study protocol, and indicate whether they approved this research or granted a formal waiver of ethical approval<br>• Include an approval number if one was obtained<br>• If the study involved *non-human primates*, add *additional details* about animal welfare and steps taken to ameliorate suffering<br>• If anesthesia, euthanasia, or any kind of animal sacrifice is part of the study, include briefly which substances and/or methods were applied<br><br>**Field Research**<br><br>Include the following details if this study involves the collection of plant, animal, or other materials from a natural setting:<br>• Field permit number<br>• Name of the institution or relevant body that granted permission	
**Data Availability**<br><br>Authors are required to make all data underlying the findings described fully available, without restriction, and from the time of publication. PLOS allows rare exceptions to address legal and ethical concerns. See the PLOS Data Policy and FAQ for detailed information.	Yes - all data are fully available without restriction

A Data Availability Statement describing where the data can be found is required at submission. Your answers to this question constitute the Data Availability Statement and **will be published in the article**, if accepted.<br><br>**Important:** Stating 'data available on request from the author' is not sufficient. If your data are only available upon request, select 'No' for the first question and explain your exceptional situation in the text box.<br><br>Do the authors confirm that all data underlying the findings described in their manuscript are fully available without restriction?	
**Describe where the data may be found in full sentences. If you are copying our sample text, replace any instances of XXX with the appropriate details.**<br><br>• If the data are **held or will be held in a public repository**, include URLs, accession numbers or DOIs. If this information will only be available after acceptance, indicate this by ticking the box below. For example: *All XXX files are available from the XXX database (accession number(s) XXX, XXX.).*<br>• If the data are all contained **within the manuscript and/or Supporting Information files**, enter the following: *All relevant data are within the manuscript and its Supporting Information files.*<br>• If neither of these applies but you are able to provide **details of access elsewhere**, with or without limitations, please do so. For example:<br><br>*Data cannot be shared publicly because of [XXX]. Data are available from the XXX Institutional Data Access / Ethics Committee (contact via XXX) for researchers who meet the criteria for access to confidential data.*<br><br>*The data underlying the results presented in the study are available from (include the name of the third party*	All relevant data are within the manuscript and its Supporting Information files.

and contact information or URL).<br>• This text is appropriate if the data are owned by a third party and authors do not have permission to share the data.<br><br>* typeset	
Additional data availability information:	Tick here if the URLs/accession numbers/DOIs will be available only after acceptance of the manuscript for publication so that we can ensure their inclusion before publication.

Powered by Editorial Manager® and ProduXion Manager® from Aries Systems Corporation

Manuscript
Click here to download Manuscript MS_Two novel species in Torulaceae.docx

Multigene phylogeny and taxonomy of *Torula hydei* and *Dendryphion hydei* spp. nov. from herbaceous litter in northern Thailand

Junfu Li[1,2,3,4], Rajesh Jeewon[6], Peter E. Mortimer[3], Mingkwan Doilom[3,5], Rungtiwa Phookamsak*[3,4,5] and Itthayakorn Promputtha*[1,2]

[1] Department of Biology, Faculty of Science, Chiang Mai University, Chiang Mai 50200, Thailand

[2] Center of Excellence in Bioresources for Agriculture, Industry and Medicine, Faculty of Science, Chiang Mai University, Chiang Mai 50200, Thailand

[3] Key Laboratory of Biodiversity and Biogeography, Kunming Institute of Botany, Chinese Academy of Sciences, Kunming 650201, P.R. China

[4] Center of Excellence in Fungal Research, Mae Fah Luang University, Chiang Rai 57100, Thailand

[5] World Agroforestry Centre, East and Central Asia, 132 Lanhei Road, Kunming 650201, P.R. China

[6] Department of Health Sciences, Faculty of Science, University of Mauritius, Reduit, Mauritius

*Corresponding Author:

itthayakorn.p@cmu.ac.th (IP)

jomjam.rp2@gmail.com (RP)

Abstract

Asexual fungi are some of the most significant microorganisms involved in decomposition of plants and contribute to nutrient recycling. During our studies on asexual fungi colonizing herbaceous litter in northern Thailand, we discovered two new fungal species, viz. *Torula hydei* and *Dendryphion hydei* spp. nov. The latter are examined, and their morphological characters are described as well as their DNA sequences from ribosomal and protein coding genes are analysed to infer their phylogenetic relationships with extant fungi. *Torula hydei* is different from other similar *Torula* species in having tiny and catenate conidia. *Dendryphion hydei* can be distinguished from other similar *Dendryphion* species in having large conidiophores and subhyaline to pale olivaceous brown, 2–4(–5)-septate conidia. Multigene phylogenetic analyses of a combined LSU, SSU, TEF1-α, RPB2 and ITS DNA sequence dataset generated from maximum likelihood and Bayesian inference analyses indicate that *T. hydei* forms a distinct lineage and basal to *T. fici*. *Dendryphion hydei* forms a distinct lineage and basal to *D. europaeum*, *D. comosum*, *D. aquaticum* and *D. fluminicola* within Torulaceae (Pleosporales, Dothideomycetes).

Keywords – ~~2~~ **new species, Dothideomycetes, Hyphomycetes, Pleosporales, Torulaceae**

Introduction

The family Torulaceae Corda was ~~validly~~ introduced by Sturm [46] and is typified by *Torula* Pers. Species in Torulaceae are known only by their asexual morphs which are characterized

by micro- or macronematous conidiophores, with or without apical branches. Conidiogenous cells are doliiform to ellipsoid or clavate, brown, smooth to verruculose, mono- to polyblastic, often cupulate. Conidia are subcylindrical, phragmosporous, acrogenous, brown, dry, smooth to verrucose, characteristically produced in branched chains [3,9,20,30,47,48]. Crous et al. [8] investigated phylogenetic relationships of this family with the inclusion of *Torula* species and accepted *Dendryphion* Wallr. and *Torula* within Torulaceae in Pleosporales. Su et al. [47] introduced *Neotorula* Ariyaw., Z.L. Luo & K.D. Hyde and two new *Dendryphion* species in Torulaceae based on molecular data. Li et al. [29] established a novel genus, *Sporidesmioides* Jun F. Li, Phook. & K.D. Hyde. Su et al. [48] examined 21 freshwater taxa in Torulaceae and updated phylogenetic relationships of taxa within the family based on ITS, LSU, TEF1-α and RPB2 genes and accommodated *Rostriconidium* Z.L. Luo, K.D. Hyde & H.Y. Su within Torulaceae. Currently, there are five accepted genera in Torulaceae viz. *Dendryphion, Neotorula*, *Rostriconidium*, *Sporidesmioides* and *Torula* [20,29,47,48].

Torula is typified by *T. herbarum* Pers. and is morphologically characterized by having terminal or lateral, monoblastic or polyblastic conidiogenous cells with a thickened and heavily melanized wall on the base and thin-walled and frequently collapsing and becoming coronate on the apex [6]. Crane and Schoknecht [7] provided details of conidiogenesis in *Torula* based on light and transmission electron microscopy. Based on their examination, conidiogenesis has provided good taxonomic insights useful to segregate *Torula* and these were also observed by Mason [33], Hughes [19], Subramanian [49] and Ellis [14,15]. However, there was little information regarding the phylogenetic relationships of *Torula* until the studies of Crous et al. [8], Li et al. [30] and Su et al. [47,48]. Based on the LSU rDNA sequence analysis, Crous et al.

[8] reported two new species, *T. fici* Crous [as '*ficus*'] and *T. hollandica* Crous. Li et al. [30] introduced four new species, *T. chiangmaiensis* Jun F. Li, Phook. & K.D. Hyde, *T. chromolaenae* Jun F. Li, Phook., Mapook & K.D. Hyde, *T. mackenziei* Jun F. Li, Phook. & K.D. Hyde and *T. pluriseptata* Jun F. Li, Phook., Camporesi & K.D. Hyde based on the analysis of a combined LSU, SSU, TEF1-α and RPB2 sequence dataset. Su et al. [48] introduced *T. aquatica* Z.L. Luo, K.D. Hyde, X.J. Su & H.Y. Su based on phylogenetic analyses of the combined ITS, LSU, RPB2 and TEF1-α sequence data. Hyde et al. [22] introduced *T. breviconidiophora* C.G. Lin & K.D. Hyde and *T. polyseptata* C.G. Lin & K.D. Hyde based on the analysis of the combined ITS, LSU, SSU and TEF1-α sequence data. To date, only 15 species have their DNA sequence data being analysed to reveal their phylogenetic placements in Torulaceae [21,22,29,30,47,48,52].

Dendryphion Wallr. was introduced by Wallroth [56] to accommodate hyphomycetous species, *D. comosum* Wallr. The genus is commonly known to be saprobic on dead stems of herbaceous plants and decaying wood, and is characterized by having erect, solitary, branched in upper part, polytretic conidiophores, forming septate, pigmented, thick-walled, finely roughened stipe and a distinct conidiogenous apparatus, with dark scars and catenate, in simple or branched chains of brown, septate (didymo- or cheiro) conidia [8,48]. Crous et al. [9] introduced *D. europaeum* Crous & R.K. Schumacher based on morphological characteristics and molecular data and later Crous et al. [8] accommodated the species in Torulaceae and further accepted *Dendryphion* in Torulaceae. Su et al. [47] circumscribed genera of Torulaceae from freshwater habitats and introduced two *Dendryphion* species, *D. aquaticum* Hong Y. Su & K.D. Hyde and *D. submersum* Hong Y. Su & K.D. Hyde and designated a reference specimen

of *D. nanum* (Nees) S. Hughes based on molecular phylogeny. Su et al. [48] also introduced *D. fluminicola* Z.L. Luo, D.J. Bhat & K.D. Hyde. Only seven *Dendryphion* species have DNA sequence data and their phylogenetic affinities to members of the Torulaceae have been investigated.

In this study, a novel *Torula* species was isolated from herbaceous litters collected from northern Thailand. Among collected samples, *Dendryphion hydei* is also recovered as a new species from northern Thailand. These species are described and illustrated. In addition, an updated phylogenetic tree with our new taxon for the family Torulaceae is provided in this paper.

Material and Methods

Isolation and identification

The specimens were collected from herbaceous litters (*Chromolaena odorata* Linn. and *Bidens pilosa* Linn.) in northern Thailand during the year 2015 to 2016. Samples were returned to the laboratory (Center of Excellence in Fungal Research, Mae Fah Luang University, Chiang Rai, Thailand) for examination and description of morphological characteristics. The specimens were observed under a Motic SMZ 168 series dissecting stereomicroscope. The conidial structures were picked up by a sterilized surgical needle and transferred into 10% lacto-glycerol on a clean slide and examined under a Nikon Eclipse 80i compound microscope and photo-captured with a Canon 600D digital camera using DIC microscopy. Macro- morphological structures were photographed with a Discovery V.8 stereo microscope fitted with a CARL ZEISS Axio Cam ERc5S microscope camera. Tarosoft® Image Frame Work program v.0.9.0.7

and Adobe Photoshop CS5 Extended version 10.0 software (Adobe Systems Inc., The United States) were used for measurements and drawing photographic plates.

Single conidia isolation was carried out to obtain pure cultures as described in Dai et al. [11]. Germinating conidia were transferred aseptically to potato dextrose agar (PDA) and malt extract agar (MEA) plates and grown at 16–30 °C in alternating day and night light. Colony characters were observed and recorded after one week and at weekly intervals [4, 5].

The type specimens were deposited in the herbarium of Mae Fah Luang University (MFLU), Chiang Rai, Thailand and the Herbarium of Cryptogams Kunming Institute of Botany Academia Sinica (KUN-HKAS), Yunnan, China. Ex-type living cultures are deposited in Mae Fah Luang University Culture Collection (MFLUCC 18-0250 and MFUCC 18-0236) and Kunming Institute of Botany Culture Collection (KUMCC 16-0037 and KUMCC 18-0009). Faces of Fungi and Index Fungorum numbers are registered as outlined in Jayasiri et al. [24] and Index Fungorum [23]. New species are established based on guidelines of Jeewon and Hyde [26].

DNA extraction, PCR amplification and sequencing

Fungal mycelium was scraped off and transferred to a 1.5 ml micro-centrifuge tube using a sterilized lancet for genomic DNA extraction. The Biospin Fungus Genomic DNA Extraction Kit-BSC14S1 (BioFlux®, P.R. China) was used to extract fungal genomic DNA, following the protocols in the manufacturer's instructions.

DNA amplification was performed by polymerase chain reaction (PCR) using the following genes (ITS, LSU, SSU, RPB2 and TEF1-α). The primers ITS5 and ITS4 primer pairs were used to amplify the ITS and 5.8S regions of the rDNA gene [58]; The primers LR0R and

LR5 were used to amplify the partial ribosomal RNA for the 28S nuclear large subunit (LSU) [54]; NS1 and NS4 were used to amplify the partial ribosomal RNA for the 18S nuclear small subunit (SSU) [58]; fRPB2-5F and fRPB2-7cR were used to amplify the partial RNA polymerase second largest subunit (RPB2) [32] and EF1-983F and EF1-2218R were used to amplify the translation elongation factor 1-alpha gene (TEF1-α) [38].

The final volume of the PCR reaction was 25 µl, containing 1 µl of DNA template, 1 µl of each forward and reward primer, 12.5 µl of 2×Easy Taq PCR SuperMix (mixture of *EasyTaq*TM DNA Polymerase, dNTPs, and optimized buffer, Beijing TransGen Biotech Co., Ltd., Beijing, P.R. China) and 9.5 µl of ddH_2O. The PCR thermal cycling conditions of ITS, LSU, SSU and TEF1-α were as follows: 94 °C for 3 minutes, followed by 35 cycles of denaturation at 94 °C for 30 seconds, annealing at 55 °C for 50 seconds, elongation at 72 °C for 1 minute, and a final extension at 72 °C for 10 minutes. The PCR thermal cycle program for RPB2 was as follows: initial denaturation at 95 °C for 5 minutes, followed by 40 cycles of denaturation at 95 °C for 1 minute, annealing at 52 °C for 2 minutes, elongation at 72 °C for 90 seconds, and final extension at 72 °C for 10 minutes. Purification and sequencing of PCR fragments with PCR primers mentioned above were carried out at Shanghai Majorbio Biopharm Technology Co., Ltd, China.

Sequence alignment and phylogenetic analyses

Phylogenetic analyses were performed from single gene (LSU dataset) as well as based on a combined LSU, SSU, TEF1-α, RPB2 and ITS sequence dataset. Sequences generated from this study were analyzed with other similar sequences obtained from GenBank and those derived from recent publications [2,22,29,30,47,48] (Table 1). The single gene alignment was

performed by using MAFFT v. 7 (http://mafft.cbrc.jp/alignment/server/) [27] and manually aligned wherever necessary in MEGA version 7.0 [28]. Further analyses for the combined dataset were analyzed by maximum likelihood (ML) implemented in RAxMLGUI v.0.9b2 [42,43,44,45] and Bayesian Inference (BI) criteria [17, 18] following the methodology in Li et al. [30].

The phylogram was represented in Treeview [35] and drawn in Microsoft PowerPoint and converted to jpeg file in Adobe Photoshop version CS5 (Adobe Systems Inc., the United States). The new sequences were submitted in GenBank (Table 1). The alignment was deposited in TreeBASE [53] under the accession number 25100.

Table 1. Taxa used in the phylogenetic analysis and their corresponding GenBank numbers. The newly generated sequences are indicated in **blue bold** font, while the type strains are in **black bold** font.

Species	Culture collection/ Voucher no.	GenBank accession numbers					References
		ITS	LSU	SSU	RPB2	TEF1-α	
Arthopyrenia salicis	CBS 368.94	KF443410	AY779288	AY538333	KF443397	KF443404	[1]
Cycasicola goaensis	**MFLUCC 17-0754**	**MG828885**	**MG829001**	**MG829112**	**–**	**MG829198**	[57]
Dendryphion aquaticum	**MFLUCC 15-0257**	**KU500566**	**KU500573**	**KU500580**	**–**	**–**	[47]
Dendryphion comosum	**CBS 208.69**	**MH859293**	**MH871026**	**–**	**–**	**–**	[55]
Dendryphion europaeum	CPC 22943	KJ869146	KJ869203	–	–	–	[9]
Dendryphion europaeum	**CPC 23231**	**KJ869145**	**KJ869202**	**–**	**–**	**–**	
Dendryphion fluminicola	KUMCC 15-0321	MG208160	MG208139	–	MG207971	MG207990	[48]
Dendryphion fluminicola	DLUCC 0849	MG208161	MG208140	–	MG207972	MG207991	
Dendryphion fluminicola	**MFLUCC17-1689**	**NR_157490**	**MG208141**	**–**	**–**	**MG207992**	
Dendryphion hydei	**KUMCC 18-0009**	**MN061343**	**MH253927**	**MH253929**	–	**MH253931**	**This study**
Dendryphion nanum	HKAS84010	KU500568	KU500575	KU500582	–	–	[47]
Dendryphion nanum	HKAS84012	KU500567	KU500574	KU500581	–	–	
Dendryphion nanum	MFLUCC 16-0987	MG208156	MG208135	–	MG207967	MG207986	[48]
Dendryphion submersum	**MFLUCC15-0271**	KU500565	**KU500572**	**KU500579**	**–**	**–**	[47]
Dendryphion submersum	KUMCC15-0455	MG208159	MG208138	–	MG207970	MG207989	[48]
Hobus wogradensis	**CBS 141484**	**NR_147652**	**KX650546**	**NG_061253**	**KX650575**	**KX650521**	[25]
Liua muriformis	**KUMCC 18-0177**	**MK433599**	**MK433598**	**MK433595**	**MK426799**	**MK426798**	[36]
Neooccultibambusa chiangraiensis	**MFLUCC 12-0584**	**NR_154238**	**KU764699**	**KU712458**	**–**	**–**	[12]
Neoroussoella bambusae	**MFLUCC 11-0124**	**KJ474827**	**KJ474839**	**–**	**KJ474856**	**KJ474848**	[31]
Neotorula aquatica	**MFLUCC 15-0342**	**KU500569**	**KU500576**	**KU500583**	**–**	**–**	[47]
Neotorula submersa	**HKAS 92660**	**NR_154247**	**KX789217**	**–**	**–**	**–**	[20]
Nigrograna mackinnonii	E5202H	JK26415	KJ605422	JK264155	JK264156	JK264154	[40]

Nigrograna mackinnonii	CBS 110022	KF015653	KF015609	GQ387553	KF015704	KF407985	[1]
Nigrograna mackinnonii	**CBS 674.75**	**NR_132037**	**GQ387613**	**GQ387552**	**–**	**–**	
Nigrograna marina	**CY 1228**	**–**	**GQ925848**	**GQ925835**	**GU479823**	**GU479848**	[50]
Occultibambusa bambusae	**MFLUCC 13-0855**	**KU940123**	**KU863112**	**KU872116**	**KU940170**	**KU940193**	[11]
Ohleria modesta	WU 36870	KX650562	–	–	KX650582	KX650533	[25]
Ohleria modesta	CBS 141480	KX650563	–	KX650513	KX650583	KX650534	
Parathyridaria ramulicola	**CBS 141479**	**NR_147657**	**KX650565**	**KX650514**	**KX650584**	**KX650536**	[25]
Parathyridaria percutanea	**CBS 868.95**	**NR_147631**	**NG_058022**	**NG_062999**	**KF366452**	**KF407987**	[1]
Parathyridaria robiniae	**MFLUCC 14-1119**	**KY511142**	**KY511141**	**–**	**–**	**KY549682**	[52]
Roussoella chiangraina	**MFLUCC 10-0556**	**NR_155712**	**KJ474840**	**–**	**KJ474857**	**KJ474849**	[31]
Roussoella nitidula	**MFLUCC 11-0182**	**KJ474835**	**KJ474843**	**–**	**KJ474859**	**KJ474852**	[31]
Roussoella scabrispora	MFLUCC 11-0624	KJ474836	KJ474844	–	KJ474860	KJ474853	[31]
Rostriconidium aquaticum	KUMCC 15-0297	MG208165	MG208144	–	MG207975	MG207995	[48]
Rostriconidium aquaticum	**MFLUCC 16-1113**	**MG208164**	**MG208143**	**–**	**MG207974**	**MG207994**	
Roussoellopsis macrospora	**MFLUCC 12-0005**	**KJ739604**	**KJ474847**	**KJ739608**	**KJ474862**	**KJ474855**	[31]
Roussoellopsis tosaensis	KT1659	–	AB524625	AB524484	AB539104	AB539117	[51]
Sporidesmium australiense	HKUCC 10833	–	DQ408554	–	DQ435080	–	[41]
Sporidesmioides thailandica	**MFLUCC 13-0840**	**MN061347**	**NG_059703**	**NG_061242**	**KX437761**	**KX437766**	[29]
Sporidesmioides thailandica	KUMCC 16-0012	MN061348	KX437758	KX437760	KX437762	KX437767	
Thyridaria broussonetiae	**CBS 141481**	**NR_147658**	**KX650568**	**NG_063067**	**KX650586**	**KX650539**	[25]
Thyridaria broussonetiae	CBS 121895	KX650567	KX650567	–	KX650585	KX650538	
Thyridariella mahakashae	**NFCCl 4215**	**MG020435**	**MG020438**	**MG020441**	**MG020446**	**MG023140**	[13]
Thyridariella mangrovei	**NFCCl 4213**	**MG020434**	**MG020437**	**MG020440**	**MG020445**	**MG020443**	[13]
Torula acaciae	**CPC 29737**	**NR_155944**	**NG_059764**	**–**	**KY173594**	**–**	[10]
Torula aquatica	DLUCC 0550	MG208166	MG208145	–	MG207976	MG207996	[48]
Torula aquatica	**MFLUCC16-1115**	**MG208167**	**MG208146**	**–**	**MG207977**	**–**	
Torula breviconidiophora	**KUMCC 18-0130**	**MK071670**	**MK071672**	**MK071697**	**–**	**MK077673**	[22]
Torula chiangmaiensis	**KUMCC 16-0039**	**MN061342**	**KY197856**	**KY197863**	**–**	**KY197876**	[30]

Torula chromolaenae	**KUMCC 16-0036**	**MN061345**	**KY197860**	**KY197867**	**KY197873**	**KY197880**	[30]
Torula fici	**CBS 595.96**	**KF443408**	**KF443385**	**KF443387**	**KF443395**	**KF443402**	[8]
Torula fici	KUMCC 15-0428	MG208172	MG208151	–	MG207981	MG207999	[48]
Torula fici	KUMCC 16-0038	MN061341	KY197859	KY197866	KY197872	KY197879	[30]
Torula gaodangensis	**MFLUCC 17-0234**	**MF034135**	**NG_059827**	**NG_063641**	–	–	[21]
Torula goaensis	**NFCCL 4040**	**NR_159045**	**NG_060016**	–	–	–	[37]
Torula herbarum	**CPC 24414**	**KR873260**	**KR873288**	–	–	–	[8]
Torula hollandica	**CBS 220.69**	**NR_132893**	**NG_064274**	**KF443389**	**KF443393**	**KF443401**	[8]
Torula hydei	**KUMCC 16-0037**	**MN061346**	**MH253926**	**MH253928**	–	**MH253930**	**This study**
Torula mackenziei	**MFLUCC 13-0839**	**MN061344**	**KY197861**	**KY197868**	**KY197874**	**KY197881**	[30]
Torula masonii	**CBS 245.57**	**NR_145193**	**NG_058185**	–	–	–	[8]
Torula masonii	DLUCC 0588	MG208173	MG208152	–	MG207982	MG208000	[47]
Torula masonii	KUMCC 16-0033	MN061339	KY197857	KY197864	KY197870	KY197877	[30]
Torula pluriseptata	**MFLUCC 14-0437**	**MN061338**	**KY197855**	**KY197862**	**KY197869**	**KY197875**	[30]
Torula polyseptata	**KUMCC 18-0131**	**MK071671**	**MK071673**	**MK071698**	–	**MK077674**	[22]
Torula sp.	CBS 246.57	KF443411	KR873290	–	–	–	[8]
Torula sp.	KUMCC 19-0112	MN507400	MN507402	MN507401	MN507404	MN507403	In prep.

Abbreviations: CBS: Westerdijk Fungal Biodiversity Institute, Utrecht, The Netherlands; **CPC**: Collection of Pedro Crous housed at CBS; **DLUCC**: Dali University Culture Collecting Center, Dali, Yunnan, China. **HKAS**: Herbarium of Cryptogams Kunming Institute of Botany Academia Sinica (HKAS), Yunnan, China; **HKUCC**: University of Hong Kong Culture Collection, Department of Ecology and Biodiversity, Hong Kong, China; **KUMCC**: Kunming Institute of Botany Culture Collection, Chinese Science Academy, Kunming, China; **MFLUCC**: Mae Fah Luang University Culture Collection, Chiang Rai, Thailand; **NFCCI**: National Fungal Culture Collection of India; **KT**: K. Tanaka.

Nomenclature

The electronic version of this article in Portable Document Format (PDF) in a work with an ISSN or ISBN will represent a published work according to the International Code of Nomenclature for algae, fungi, and plants, and hence the new names contained in the electronic publication of a PLOS ONE article are effectively published under that Code from the electronic edition alone, so there is no longer any need to provide printed copies.

In addition, new names contained in this work have been submitted to Index Fungorum from where they will be made available to the Global Names Index. The unique Index Fungorum number can be resolved and the associated information viewed through any standard web browser by appending the Index Fungorum number contained in this publication to the prefix www.indexfungorum.org/. The online version of this work is archived and available from the following digital repositories: [INSERT NAMES OF DIGITAL REPOSITORIES WHERE ACCEPTED MANUSCRIPT WILL BE SUBMITTED (PubMed Central, LOCKSS etc)]. All PLOS ONE articles are deposited in PubMed Central and LOCKSS. If your institute, or those of your co-authors, has its own repository, we recommend that you also deposit the published online article there and include the name in your article. A complete explanation of our guidelines for publishing new species can be found on our website: http://www.plosone.org/static/guidelines#fungal

Compliance with Ethical Standards

There is no conflict of interest (financial or non-financial) and all authors have agreed to submission of paper. The authors also declare that they have no conflict of interest and confirm

that the field studies did not involve endangered or protected species.

Results

Phylogenetic analyses

The combined LSU, SSU, TEF1-α, RPB2 and ITS sequence dataset comprises 65 taxa with *Occultibambusa bambusae* (MFLUCC 13-0855) and *Neooccultibambusa chiangraiensis* (MFLUCC 12-0559) as the outgroup taxa. Bayesian Inference (BI) and maximum likelihood (ML) analyses of the combined dataset were performed to determine the placement of our new taxa and infer relationships at the intrageneric level as well as resolving the phylogenetic relationships of the core families in Pleosporales. The phylogenetic trees obtained from BI and ML analyses resulted in trees with largely similar topologies and also similar to those generated from previous studies based on maximum likelihood analysis [21,30,48]. The best scoring RAxML tree is shown in Figure 1, with the final ML optimization likelihood value of -31463.916972 (ln). The dataset consists of 4053 total characters including gaps (LSU: 1–840 bp, SSU: 841–1776 bp, TEF1-α: 1777–2566 bp, RPB2: 2567–3418 bp，ITS: 3419–4053). RAxML analysis yielded 1568 distinct alignment patterns and 32.43% of undetermined characters or gaps. Estimated base frequencies were as follows: A = 0.246541, C = 0.258447, G = 0.270790, T = 0.224222, with substitution rates AC = 1.436632, AG = 3.543120, AT = 1.440155, CG = 0.960003, CT = 6.670420, GT = 1.000000. The proportion of invariable sites I = 0, the gamma distribution shape parameter alpha = 0.180447 and the Tree-Length = 3.140857. Bayesian posterior probabilities (BYPP) from MCMC were evaluated with final average standard deviation of split frequencies = 0.008264.

Most of the core genera of Torulaceae and other representative genera in Nigrogranaceae, Ohleriaceae, Roussoellaceae and Thyridariaceae are included in our phylogenetic analysis (Fig. 1). Torulaceae formed a well-resolved clade (100% ML and 1.00 PP) with a close relationship to Roussoellaceae and Thyridariaceae. Species of different genera currently accommodated in Torulaceae formed well-resolved subclades except with *Sporidesmioides* which is recovered as basal to other genera with significant Bayesian support (1.00 PP) but with low support in ML analysis (48% ML, data not shown). *Torula* is recovered as a strongly monophyletic genus in Torulaceae. *Torula hydei* is sister to *T. fici* with high support (100% ML and 1.00 PP). *Dendryphion hydei* forms a distinct lineage and related to *D. europaeum*, *D. comosum*, *D. aquaticum*, *D. fluminicola* and *D. submersum* with significant support in BI analysis (0.95 PP).

Fig. 1 Phylogenetic construction using RAxML-based analysis of a combined LSU, SSU, TEF1-α, RPB2 and ITS DNA sequence dataset. Bootstrap support values for maximum likelihood (ML) equal to or greater than 70% and Bayesian posterior probabilities (PP) equal to or greater than 0.95 are shown as "ML/PP" above the nodes. The tree is rooted to *Occultibambusa bambusae* (MFLUCC 13-0855) and *Neooccultibambusa chiangraiensis* (MFLUCC 12-0559). The type strains are in black bold and the newly generated sequences are indicated in blue bold.

Taxonomy

Dendryphion hydei J.F. Li, Phookamsak & Jeewon, *sp. nov.* Fig. 2

[urn:lsid:indexfungorum.org:names:556746]

Facesoffungi number: FOF0457

Etymology – Named in honour of Kevin D. Hyde for his excellent contribution to mycology and on his 65th birthday celebration.

Holotype – KUN-HKAS 97502

Saprobic on a branch litter of *Bidens pilosa* Linn. (Asteraceae). **Sexual morph**: Undetermined. **Asexual morph**: *Colonies* on the substratum superficial, effuse, gregarious, hairy, brown to dark brown. *Mycelium* composed of branched, septate, pale brown to brown hyphae. *Conidiophores* 260–380 µm long × 7–14 µm diam. (13–17 µm diam. at the base) ($\bar{x}$ = 356.7 × 9.9 µm, n = 10) macronematous, mononematous, septate, verrucose, thick-walled, branching simple or penicillate at the tip of primary branches, brown, flexuous. *Conidiogenous cells* 6–10 µm long × 3–5 µm diam. ($\bar{x}$ = 8 × 3.8 µm, n = 20) terminal, integrated, pale brown, polytretic. *Conidia* (17–)20–30(–35) µm long × 4–7 µm diam. ($\bar{x}$ = 26.5 × 5.6 µm, n = 30) single, subhyaline to pale olivaceous brown, slightly paler at the end cells, dry, verrucose, monilioid, 2–4(–5)-septate, constricted at the septa. Conidial secession schizolytic.

Cultural characteristics: Conidia germinating on PDA within 14 hours and germ tubes produced from the apex. Colonies growing on PDA, reaching 5 cm in 21 days at 16–30 °C, mycelium partly superficial, partly immersed, slightly effuse, hairy, vertical, with regular edge, white to grayish-brown, not produced pigmentation on media agar.

Material examined: THAILAND, Chiang Mai Province, Mae Taeng District, Mushroom Research Centre, on a branch litter of *Bidens pilosa* Linn., 12 July 2016, J.F. Li, FHP3 (HKAS 97502, **holotype**), ex-type living culture, MFLUCC 18-0236, KUMCC 18-0009.

Notes – *Dendryphion hydei* resembles *D. aquaticum* and *D. europaeum* in morphology.

However, these species can be distinguished based on the size of the conidiophores, conidiogenous cells and conidia, as well as the conidial septation and habitats (see Table 2). *Dendryphion hydei* has 2–4(–5)-septate conidia and inhabit in a terrestrial environment, similar to *D. europaeum*. However, *D. europaeum* has smaller conidiophores and conidia, and the conidia of *D. europaeum* are (2–)3(–5)-septate while *D. aquaticum* inhabit in a freshwater environment and has 3–6-septate conidia [9,47]. In the phylogenetic tree, *D. hydei* forms a separate lineage and clustered with *D. europaeum*, *D. comosum*, *D. aquaticum* and *D. fluminicola* with significant support in Bayesian inference analysis (0.95 PP). In this study, we collected *D. hydei* from *Bidens pilosa*, which is a new host record for this species. A morphometric comparison of the new taxon with other similar taxa of *Dendryphion* provide in Table 2.

Fig. 2 *Dendryphion hydei* (HKAS 97479, holotype) a Colonies on branch of *Bidens pilosa*. b, c Apex of conidiophores with conidial structures. d, e Conidiophores. f–i Conidiogenous cells. j–q Conidia. Scale bars: a = 100 µm, d, e = 50 µm, b, f–i = 20 µm, b, c, f–q = 10 µm.

Table 2 Synopsis of morphological features of *Dendryphion* species discussed in this study

Species	Size (μm)			Conidial septation	Host/substrate and habitat	Distribution	Reference
	Conidiophores	Conidiogenous cells	Conidia				
Dendryphion aquaticum	250–285 × 7.5–11.5	5–9 × 4–6	22–33 × 6.5–7.5	3–6	Decaying wood submerged in stream	China (Yunnan)	[47]
Dendryphion comosum	Up to 400 × 9–14	Up to16 × 5–8	9–65 × 5–9	1–5(−9)	Various hosts and substrates	Cosmopolitan distribution	[16, 39]
Dendryphion europaeum	180–250 × 8–10	6–10 × 5–7	(15–)20–28(–33) × (6–)7	(2–)3(–5)	*Hedera helix*, *Heracleum sphondylium*	Germany, Netherlands	[9]
Dendryphion fluminicola	114–176 × 7–10	N/A	31–46 × 8–9	2–6	Decaying wood submerged in a stream in Cangshan Mountain, Lancang River and Jinsha River	China (Yunnan)	[48]
Dendryphion hydei	260–380 × 7–14	6–10 × 3–5	(17–)20–30(–35) × 4–7	2–4(–5)	Branch litter of of *Bidens pilosa*	Thailand	This study
Dendryphion nanum	52–64 × 6.5–8.5	13–19 × 6–8	56.7–74.5 × 10–12	3–11	Various hosts and substrates	Cosmopolitan distribution	[16,47]
Dendryphion submersum	210–335 × 3.5–4.5	11–15 × 4.5–6.5	15–25 × 5–7	2–5	Decaying wood submerged in stream	China (Yunnan)	[47]

Torula hydei J.F. Li, Phookamsak & Jeewon, *sp. nov.* Fig. 3

[urn:lsid:indexfungorum.org:names:556747]

Facesoffungi number: FoF 04573

Etymology – Named in honour of Kevin D. Hyde for his excellent contribution to mycology and on his 65th birthday celebration.

Holotype – HKAS 97478

Saprobic on an aerial dead branch of *Chromolaena odorata* Linn. **Sexual morph**: Undetermined. **Asexual morph**: *Colonies* discrete on host, black, powdery. *Mycelium* immersed on the substrate, composed of septate, branched, smooth, light brown hyphae. *Conidiophores* (1.5–)2–3 µm long × 1.5–2 µm diam. ($\bar{x}$ = 2.2 × 1.8 µm, n = 10), macronematous, mononematous, solitary, erect, light brown, verruculose, thick-walled, consist of one cell or reduced to conidiogenous cells, without apical branches, subcylindrical to subglobose, arising from hyphae. *Conidiogenous cells* 3–5.5 µm long × 4.3–5 µm diam. ($\bar{x}$ = 3.8 × 4.5 µm, n = 20), polyblastic, terminal, dark brown to black, smooth to minutely verruculose, thick-walled, doliiform to ellipsoid. (7.5–)8–14 µm long × 2–4 µm diam.($\bar{x}$ = 10.4 × 3.4 µm, n = 30), solitary to catenate, acrogenous, simple, phragmosporous, light brown to brown, minutely verruculose, 2–3-septate, rounded at both ends, composed of subglobose cells, slightly constricted at some septa, chiefly sub-cylindrical. *Conidial secession* schizolytic.

Cultural characteristics: Conidia germinating on PDA within 14 hours and germ tubes produced from the apex. Colonies growing on PDA, reaching 5 cm in 10 days at 16–30 °C, mycelium partly superficial, partly immersed, slightly effuse, hairy, vertical, with regular edge, light brown to brown, not produced pigmentation on media agar; not sporulated on media agar

within 2 months.

Material examined: THAILAND, Chiang Mai Province, Mae Taeng District, on an aerial dead branch of *Chromolaena odorata* Linn. (Asteraceae), 26 December 2015, J.F. Li, MRC2 (HKAS 97478, **holotype**), ex-type living culture, MFLUCC 18-0250, KUMCC 16-0037.

Notes – *Torula hydei* resembles *T. herbarum* and *T. fici* in having 2–3-septate, catenated, brown, verruculose conidia, but differs in having smaller conidia [9]. Phylogenetic analyses showed that *T. hydei* constitutes an independent lineage basal to *T. fici* (100% ML and 1.00 BYPP).

Fig. 3 *Torula hydei* (HKAS 97478, holotype). a Colonies on dead branch of Chromolaena odorata. b–e Conidiophores with conidiogenous cell. f–j Budding on conidia. k, l Conidia in chain. m–t Conidia. Scale bars: a = 100 µm, b, k–l = 5 µm, c, f–j, q–t = 2 µm, d, e, m–p = 1 µm

Discussion

Taxonomic characterizations of taxa in Torulaceae have been well-studied since Crous et al. [8] re-classified *Torula* and *Dendryphion* in Torulaceae (Pleosporales, Dothideomycetes) based on phylogenetic analyses of LSU sequence data. Subsequent authors introduced the new genera and species in this family based on multigene phylogenetic analyses coupled with morphological characteristics (see Table 3) [21, 29, 30, 47, 48, 52]. However, there are more than 520 epithets under the genus *Torula* and 85 epithets under *Dendryphion* available in Index Fungorum [23], but these described species lack DNA sequence data to verify their

phylogenetic placement and affinities with other related fungi. Nevertheless, many species previously described as *Torula* and *Dendryphion* have also been synonymized to many genera in Sordariomycetes [23]. Taxa in these genera need to be clarified based on molecular data.

Torula and *Dendryphion* are widespread on hosts and habitats and commonly found as saprobes in both terrestrial and aquatic habitats from temperate to tropical regions [9,16,21,29, 30,47,48,52]. In this study, species in Torulaceae collected from herbaceous plants in northern Thailand were examined. Our new taxon, *Torula hydei* is characterized by morphs that correspond to those outlined by Li et al. [30]. However, *T. hydei* is unique in having very tiny conidia as compared to other similar species. We also note distinct nucleotide base pair differences between *T. hydei* and *T. fici* across TEF1-α gene region analysed (43/760 bp, 5.7% difference).

Dendryphion hydei is unique in having large conidiophores and subhyaline to pale olivaceous brown, 2–4(–5)-septate conidia to compare with other related species in *Dendryphion* (Table 2). Our multiloci phylogeny also positions our new taxon as independent lineage and phylogenetically apart from other species (Fig. 1). A comparison of TEF1-α nucleotides shows that *Dendryphion hydei* differs from *D. fluminicola* in 20/852 bp (2.3% difference), from *D. submersum* in 30/902 bp (3.3% difference). A comparison of ITS nucleotides shows that *D. hydei* differs from *D. europaeum* in 19/553 bp (3.4% difference) and differs from *D. aquaticum* in 6/398 bp (1.5% difference). Phylogenetic analyses support *D. hydei* as a new species in *Dendryphion*. These tally with recommendations outlined by Jeewon and Hyde [26] to establish our new species.

It is interesting to note that species of Torulaceae have been found to be mostly associated

with the host family Asteraceae. In this study our new strains were collected from Asteraceae and Li et al. [30] also reported two novel *Torula* species, *T. chromolaenae* and *T. mackenziei* from Asteraceae, indicating that Asteraceae harbors a diversity of these fungi. *Dendryphion hydei* collected from an herbaceous host collected in northern Thailand is also the first record on the host (*Bidens pilosa*) and location. Our combined LSU, SSU, TEF1-α, RPB2 and ITS phylogenetic analyses also support *D. hydei* as a new species.

Table 3 Synopsis of morphological features of the genera in Torulaceae.

Genus	Morphological features			Reference
	**Conidia**	**Conidiophores**	**Conidiogenous cells**	
Dendryphion	Acropleurogenous, catenate or solitary, simple or branched, cylindrical to obclavate, or cheiroid, pale to mid brown or olivaceous brown, multi-septate, smooth or verrucose	Macronematous, mononematous, branched at the apex, brown to black, smooth or with verruculose at the upper part, with paler branches	Mono- or polytretic, integrated, terminal and intercalary on branches, sympodial, clavate, cylindrical or doliiform, cicatrized, with large and dark scars.	[47,48]
Neotorula	Acrogenous, in chains, clavate to subcylindrical, septate, dark bands at the septa, pale green when young, brown when mature, verruculose	Macronematous, mononematous, cylindrical, 3–6-septate, with one or severa short branches near the apex, smooth, dark brown, paler towards the apex,	Tretic, with a distinct pore, integrated, terminal, pale brown or subhyaline, doliiform or lageniform	[47]
Rostriconidium	Solitary, pyriform to rostrate, dark brown to black, with a thick, black truncate scar at the base and pale pigment cell above the scar, narrowly cylindrical and obtuse at the apex	Macronematous, mononematous, single or caespitose, septate, smooth, brown or dark brown, unbranched, thick-walled, cylindrical, arising from a stromatic base.	Monotretic or polytretic, integrated, terminal, cylindrical, dark brown	[48]
Sporidesmioides	Acrogenous, solitary, pyriform to rostrate, ampulliform to obclavate, truncate at the base, septate, brown to dark brown, with paler at the upper end cells, smooth or verruculose to echinulate	Macronematous, mononematous, scattered, unbranched, straight to curved, sometimes percurrently proliferating	Polyblastic, integrated, indeterminate or percurrent, terminal, sometimes intercalary sympodial, dark and prominent, cylindrical or doliiform.	[29]
Torula	Acrogenous, in branched chains, subcylindrical to cylindrical, brown, constricted at septa, smooth to verrucose, conidial cells subglobose	Micronematous, reduced to conidiogenous cells, or with a brown supporting cell	Mono- to polyblastic, solitary on mycelium, doliiform to ellipsoid or clavate, cupulate, brown, smooth to verruculose,	[8,30,47]

Author Contributions

Conceptualization: Rajesh Jeewon, Rungtiwa Phookamsak, Junfu Li.

Data curation: Junfu Li.

Formal analysis: Junfu Li, Rungtiwa Phookamsak, Rajesh Jeewon.

Funding acquisition: Itthayakorn Promputtha, Rungtiwa Phookamsak.

Investigation: Junfu Li, Rungtiwa Phookamsak, Mingkwan Doilom, Rajesh Jeewon.

Methodology: Junfu Li, Rungtiwa Phookamsak

Project administration: Rungtiwa Phookamsak

Supervision: Peter E. Mortimer, Rajesh Jeewon, Itthayakorn Promputtha.

Writing – original draft: Junfu Li, Rungtiwa Phookamsak, Rajesh Jeewon, Mingkwan Doilom.

Writing – review & editing: Itthayakorn Promputtha, Rajesh Jeewon, Peter E. Mortimer.

Acknowledgements

The authors are grateful to the Mushroom Research Foundation, Chiang Mai, Thailand and Key Research Program of Frontier Sciences of the Chinese Academy of Sciences (grant no. QYZDY-SSW-SMC014) for supporting this research. We also acknowledge that Biology Experimental Center, Germplasm Bank of Wild Species, Kunming Institute of Botany, Chinese Academy of Sciences provide molecular laboratory facilities for molecular work. Rungtiwa Phookamsak thanks CAS President's International Fellowship Initiative (PIFI) for young staff (grant no. Y9215811Q1) and National Science Foundation of China (NSFC) project code 31850410489 (grant no. Y81I982211) for financial support. Itthayakorn Promputtha grateful

to thank Chiang Mai University for partially support of this research work. Rajesh Jeewon would like to thank Mae Fah Luang University for giving him the opportunity as a visiting professor to the Center of Excellence in Fungal Research and University of Mauritius for research support. Peter E Mortimer would like to thank the National Science Foundation of China and the South East Asian Biodiversity resources Institute, Chinese Academy of Sciences, for financial support under the following grants: 41761144055, 41771063, Y4ZK111B01. Mingkwan Doilom would like to thank the 5th batch of Postdoctoral Orientation Training Personnel in Yunnan Province and the 64th batch of China Postdoctoral Science Foundation. Jun-Fu Li thanks to Emeritus Prof. Kevin D. Hyde, Dr. Shaun Pennycook, Dr. Dhanushka Wanasinghe, Hong-Bo Jiang, Dr. Zonglong Luo for their available suggestions and help.

References

1. Ahmed SA, van de Sande WWJ, Stevens DA, Fahal, A, van Diepeningen AD, Menken SBJ, de Hoog GS. Revision of agents of black-grain eumycetoma in the order Pleosporales. Persoonia. 2014; 33(1):141–154. https://doi: 10.3767/003158514X684744
2. Ariyawansa HA, Hyde KD, Jayasiri SC, Buyck B, Chethana KWT, Dai DQ, et al. Fungal diversity notes 111–252—Taxonomic and phylogenetic contributions to fungal taxa. Fungal Divers. 2015; 75: 27–274. http://dx.doi.org/10.1007/s13225-015-0355-4
3. Bhat DJ. Fascinating Microfungi (Hyphomycetes) Of Western Ghats-India. Department of Botany. Goa University. 2017.
4. Boehm EWA, Mugambi GK, Miller AN, Huhndorf SM, Marincowitz S, Spatafora JW, et al. A molecular phylogenetic reappraisal of the Hysteriaceae, Mytilinidiaceae and

Gloniaceae (Pleosporomycetidae, Dothideomycetes) with keys to world species. Stud Mycol. 2009; 64: 49–83. http://doi: 10.3114/sim.2009.64.03

5. Chomnunti P, Hongsanan S, Aguirre-Hudson B, Tian Q, Peršoh D, Dhami MK, et al. The sooty moulds. Fungal Divers. 2014; 66: 1–36. http://doi:10.1007/s13225-014-0278-5

6. Crane JL, Miller AN. Studies in genera similar to *Torula*: *Bahusaganda*, *Bahusandhika*, *Pseudotorula*, and *Simmonsiella* gen. nov. IMA Fungus. 2016; 7(1): 29–45. http://doi: 10.5598/imafungus.2016.07.01.03

7. Crane JL, Schoknecht JD. Revision of *Torula* species. *Rutola*, a new name for *Torula graminis*. Can J Bot. 1977; 55: 3013–3019. http:// doi: 10.1139/b77-339

8. Crous PW, Carris LM, Giraldo A, Groenewald JZ, Hawksworth DL, Hernández-Restrepo M, et al. The Genera of Fungi-fixing the application of the type species of generic names–G2: *Allantophomopsis, Latorua, Macrodiplodiopsis, Macrohilum, Milospium, Protostegia, Pyricularia, Robillarda, Rotula, Septoriella, Torula,* and *Wojnowicia*. IMA Fungus. 2015; 6(1): 163–198. http://doi: 10.5598/imafungus.2015.06.01.11

9. Crous PW, Shivas RG, Quaedvlieg W, van der Bank M., Zhang Y, Summerell BA, et al. Fungal Planet description sheets: 214–280. Persoonia. 2014; 32(1):1 84–306. http://doi: 10.3767/003158514X682395

10. Crous PW, Wingfield MJ, Burgess TI, Hardy GE, Crane C, Barrett S, et al. Fungal Planet description sheets: 469–557. Persoonia. 2016; 37: 218–403. http://doi: 10.3767/003158516X694499

11. Dai DQ, Phookamsak R, Wijayawardene NN, Li WJ, Bhat DJ, Xu JC, et al. Bambusicolous fungi. Fungal Divers. 2017; 82(1): 1–105.

12. Doilom M, Dissanayake AJ, Wanasinghe DN, Boonmee S, Liu JK, Bhat DJ, et al. Microfungi on *Tectona grandis* (teak) in northern Thailand. Fungal Divers. 2017; 82(1): 107–182. https://doi.org/10.1007/s13225-016-0368-7
13. Devadatha B, Sarma VV, Jeewon R, Wanasinghe DN, Hyde KD, Jones EG. *Thyridariella*, a novel marine fungal genus from India: morphological characterization and phylogeny inferred from multigene DNA sequence analyses. Mycol Prog. 2018; 1–14. https://doi.org/10.1007/s11557-018-1387-4
14. Ellis MB. Dematiaceous Hyphomycetes. Commonwealth Mycological Institute, Kew.1971.
15. Ellis MB. More Dematiaceous Hyphomycetes. Commonwealth Mycological Institute, Kew. 1976.
16. Farr DF, Rossman AY. Fungal Databases, U.S. National Fungus Collections, ARS, USDA. Retrieved January 3, 2019; from https://nt.ars-grin.gov/fungaldatabases/
17. Huelsenbeck JP, Ronquist F. MRBAYES: Bayesian inference of phylogenetic trees. Bioinformatics. 2001; 17:754–755. https://doi.org/10.1093/bioinformatics/17.8.754
18. Huelsenbeck JP, Ronquist F, Nielsen R, Bollback JP. Bayesian inference of phylogeny and its impact on evolutionary biology. Science. 2001; 294:2310–2314. https://doi: 10.1126/science.1065889
19. Hughes SJ. Conidiophores, conidia, and classification. Can J Bot. 1953; 31:577–659. https://doi.org/10.1139/b53-046
20. Hyde KD, Hongsanan S, Jeewon R, Bhat DJ, McKenzie EHC, Jones EBG, et al. Fungal diversity notes 367–490: taxonomic and phylogenetic contributions to fungal taxa. Fungal Divers. 2016; 80:1–270. https://doi.org/10.1007/s13225-016-0373-x

21. Hyde KD, Norphanphoun C, Abreu VP, Bazzicalupo A, Chethana KWT, Clericuzio M, et al. Fungal diversity notes 603–708: taxonomic and phylogenetic notes on genera and species. Fungal Divers. 2017; 87:1–235. https://doi.org/10.1007/s13225-017-0391-3
22. Hyde KD, Tennakoon DS, Jeewon R, Bhat DJ, Maharachchikumbura SSN, Rossi W, et al. Fungal diversity notes 1036–1150: taxonomic and phylogenetic contributions on genera and species of fungal taxa. Fungal Divers. 2019; 96:1–242. https://doi.org/10.1007/s13225-019-00429-2
23. Index Fungorum. 2019 http://www.indexfungorum.org/names/Names.asp (Accessed 1 February 2019).
24. Jayasiri SC, Hyde KD, Ariyawansa HA, Bhat DJ, Buyck B, Cai L, et al. The Faces of Fungi database: fungal names linked with morphology, phylogeny and human impacts. Fungal Divers. 2015; 74:3–18. https://doi.org/10.1007/s13225-015-0351-8
25. Jaklitsch WM, Voglmayr H. Hidden diversity in Thyridaria and a new circumscription of the Thyridariaceae. Stud Mycol. 2016; 85:35–64.
26. Jeewon R, Hyde KD. Establishing species boundaries and new taxa among fungi: recommendations to resolve taxonomic ambiguities. Mycosphere. 2016; 7(11):1669–1677. https://doi: 10.5943/mycosphere/7/11/4
27. Katoh K, Standley DM. MAFFT multiple sequence alignment software version 7: improvements in performance and usability. Mol Biol Evol. 2013; 30:772–780. https://doi: 10.1093/molbev/mst010
28. Kumar S, Stecher G, Tamura K. MEGA7: Molecular Evolutionary Genetics Analysis version 7.0. Mol Biol Evol. 2016; 33(7):1870-1874. https://doi: 10.1093/molbev/msw054.

29. Li JF, Bhat DJ, Phookamsak R, Mapook A, Lumyong S, Hyde KD. *Sporidesmioides thailandica* gen. et sp. nov. (Dothideomycetes) from northern Thailand. Mycol Prog. 2016; 15(10–11):1169–1178. https://doi: 10.1007/s11557-016-1238-0

30. Li JF, Phookamsak R, Jeewon R, Bhat DJ, Mapook A, Camporesi E, et al. Molecular taxonomy and morphological characterization reveal new species and new host records of *Torula* species (Torulaceae, Pleosporales). Mycol Prog. 2017; 16:447–461. https://doi: 10.1007/s11557-017-1292-2

31. Liu JK, Phookamsak R, Dai DQ, Tanaka K, Jones EBG, Xu JC, et al. *Roussoellaceae,* a new pleosporalean family to accommodate the genera *Neoroussoella* gen. nov., *Roussoella* and *Roussoellopsis*. Phytotaxa. 2014; 181(1):1−33. http://dx.doi.org/10.11646/phytotaxa.181.1.1

32. Liu YJ, Whelen S, Hall BD. Phylogenetic relationships among ascomycetes: evidence from an RNA polymerase II subunit. Mol Biol Evol. 1999; 16:1799−1808. http://doi:10.1093/oxfordjournals.molbev.a026092

33. Mason EW. Annotated account of fungi received at the Imperial Mycological Institute. List 2. Fasc. 3 (special part). Commonw. Mycol Pap. 1941; 5:1–144.

34. MycoBank. available from: http://www.mycobank.org/ (accessed: January 2019). 2019.

35. Page RDM. TreeView: an application to display phylogenetic trees on personal computers. CABIOS. 1996; 12:357–358. http://doi: 10.1093/bioinformatics/12.4.357

36. Phookamsak R, Hyde KD, Jeewon R, Bhat DJ, Gareth Jones EB, Maharachchikumbura SSN, et al. Fungal diversity notes 929–1035: taxonomic and phylogenetic contributions on genera and species of fungi. Fungal Divers. 2019; 95(1):1–273. https://doi.org/10.1007/s13225-019-00421-w

37. Pratibha J, Prabhugaonkar A. *Torula goaensis*, a new asexual ascomycetous fungus in Torulaceae. Webbia. 2017; 72(2):171–175. https://doi.org/10.1080/00837792.2017.1349237

38. Rehner SA. Primers for Elongation Factor 1-alpha (EF1-alpha). 2001. Available from: http://ocid. NACSE.ORG/research/deephyphae/EF1primer.pdf.

39. Seifert K, Morgan-Jones G, Gams W, Kendrick B. The genera of hyphomycetes. CBS-KNAW Fungal Biodiversity Centre, Utrecht. 2011.

40. Shaw JJ, Spakowicz DJ, Dalal RS, Davis JH, Lehr NA, Strobel SA. Biosynthesis and genomic analysis of medium-chain hydrocarbon production by the endophytic fungal isolate *Nigrograna mackinnonii* E5202H. Appl. Microbiol. Biotechnol. 2015; 99(8):3715–3728. https://doi: 10.1007/s00253-014-6206-5

41. Shenoy BD, Jeewon R, Wu WP, Bhat DJ, Hyde KD. Ribosomal and RPB2 DNA sequence analyses suggest that *Sporidesmium* and morphologically similar genera are polyphyletic. Mycol Res. 2006; 110(8):916–928. https://doi.org/10.1016/j.mycres.2006.06.004

42. Silvestro D, Michalak I. RAxML GUI: a graphical front–end for RAML http://sourceforge.net/projects/raxmlgui. 2010.

43. Stamatakis A. RAxML-VI-HPC: maximum likelihood-based phylogenetic analyses with thousands of taxa and mixed models. Bioinformatics. 2006; 22:2688–2690. http://doi:10.1093/bioinformatics/btl446

44. Stamatakis A. RAxML version 8: a tool for phylogenetic analysis and post-analysis of large phylogenies. Bioinformatics. 2014; 30(9):1312–1313. https://doi.org/10.1093/bioinformatics/btu033

45. Stamatakis A, Hoover P, Rougemont J. A rapid bootstrap algorithm for the RAxML web servers. Systematic Biology. 2008; 57:758–771. https://doi.org/10.1080/10635150802429642

46. Sturm J. Deutschlands Flora, Abt. III. Die Pilze Deutschlands. 1829; 2: 1–136.

47. Su HY, Hyde KD, Maharachchikumbura SSN, Ariyawansa HA, Luo ZL, Promputtha I, et al. The families Distoseptisporaceae fam. nov., Kirschsteiniotheliaceae, Sporormiaceae and Torulaceae, with new species from freshwater in Yunnan Province, China. Fungal Divers. 2016; 80(1):375–409. https://doi:10. 1007/ s13225-016-0362-0

48. Su XJ, Luo ZL, Jeewon R, Bhat DJ, Bao DF, Li WL, et al. Morphology and multigene phylogeny reveal new genus and species of Torulaceae from freshwater habitats in northwestern Yunnan, China. Mycol Prog. 2018; 17(5):531–545. https://doi.org/10.1007/s11557-018-1388-3

49. Subramanian CV. Hyphomycetes: An account of Indian species except Cercosporae. New Delhi: Indian Council of Agricultural Research. 1971.

50. Suetrong S, Schoch CL, Spatafora JW, Kohlmeyer J, Volkmann-Kohlmeyer B, Sakayaroj J, et al. Molecular systematics of the marine Dothideomycetes. Stud Mycol. 2009; 64:155–173. https://doi.org/10.3114/sim.2009.64.09

51. Tanaka K, Hirayama K, Yonezawa H, Hatakeyama S, Harada Y, Sano T, et al. Molecular taxonomy of bambusicolous fungi: *Tetraplosphaeriaceae*, a new Pleosporalean family with Tetraploa-like anamorphs. Stud Mycol. 2009; 64:175−209. https://doi.org/10.3114/sim.2009.64.10

52. Tibpromma S, Hyde KD, Jeewon R, Maharachchikumbura SSN, Liu JK, Bhat DJ, et al.

Fungal diversity notes 491–602: taxonomic and phylogenetic contributions to fungal taxa. Fungal Divers. 2017; 83:1–261. https://dio.org/10.1007/s13225-017-0378-0

53. TreeBASE. available from: https://treebase.org/ (accessed: January 2019). 2019.

54. Vilgalys R, Hester M. Rapid genetic identification and mapping of enzymatically amplified ribosomal DNA from several *Cryptococcus* species. J Bacteriol. 1990; 172:4238–4246. https://doi:10.1128/jb.172.8.4238-4246.1990

55. Vu D, Groenewald M, De Vries M, Gehrmann T, Stielow B, Eberhardt U, et al. Large-scale generation and analysis of filamentous fungal DNA barcodes boosts coverage for kingdom fungi and reveals thresholds for fungal species and higher taxon delimitation. Studi Mycol. 2019; 92:135–154.

56. Wallroth CFW. Flora Cryptogamica Germaniae. 1833; 2:1–923

57. Wanasinghe DN, Phukhamsakda C, Hyde KD, Jeewon R, Lee HB, Jones EBG, et al. Fungal diversity notes 709–839: taxonomic and phylogenetic contributions to fungal taxa with an emphasis on fungi on *Rosaceae*. Fungal Divers. 2018; 89:1–236. https://doi.org/10.1007/s13225-017-0378-0

58. White TJ, Bruns T, Lee SJWT, Taylor JW. Amplification and direct sequencing of fungal ribosomal RNA genes for phylogenetics. PCR - Protocols and Applications. 1990; 18:315–322.

Supporting Information

Fig. 1 Phylogenetic construction using RAxML-based analysis of a combined LSU, SSU, TEF1-α, RPB2 and ITS DNA sequence dataset. Bootstrap support values for maximum

likelihood (ML) equal to or greater than 70 % and Bayesian posterior probabilities (PP) equal to or greater than 0.95 are shown as “ML/PP” above the nodes. The tree is rooted to *Occultibambusa bambusae* (MFLUCC 13-0855) and *Neooccultibambusa chiangraiensis* (MFLUCC 12-0559). The type strains are in black bold and the newly generated sequences are indicated in blue bold.

Fig. 2 *Dendryphion hydei* (HKAS 97479, holotype) a Colonies on branch of *Bidens pilosa*. b, c Apex of conidiophores with conidial structures. d, e Conidiophores. f–i Conidiogenous cells. j–q Conidia. Scale bars: a = 100 µm, d, e = 50 µm, b, f–i = 20 µm, b, c, f–q = 10 µm

Fig. 3 *Torula hydei* (HKAS 97478, holotype). a Colonies on dead branch of Chromolaena odorata. b–e Conidiophores with conidiogenous cell. f–j Budding on conidia. k, l Conidia in chain. m–t Conidia. Scale bars: a = 100 µm, b, k–l = 5 µm, c, f–j, q–t = 2 µm, d, e, m–p = 1 µm

Fig. 1 Phylogenetic tree

Click here to download Figure Fig. 1 ML tree.jpg

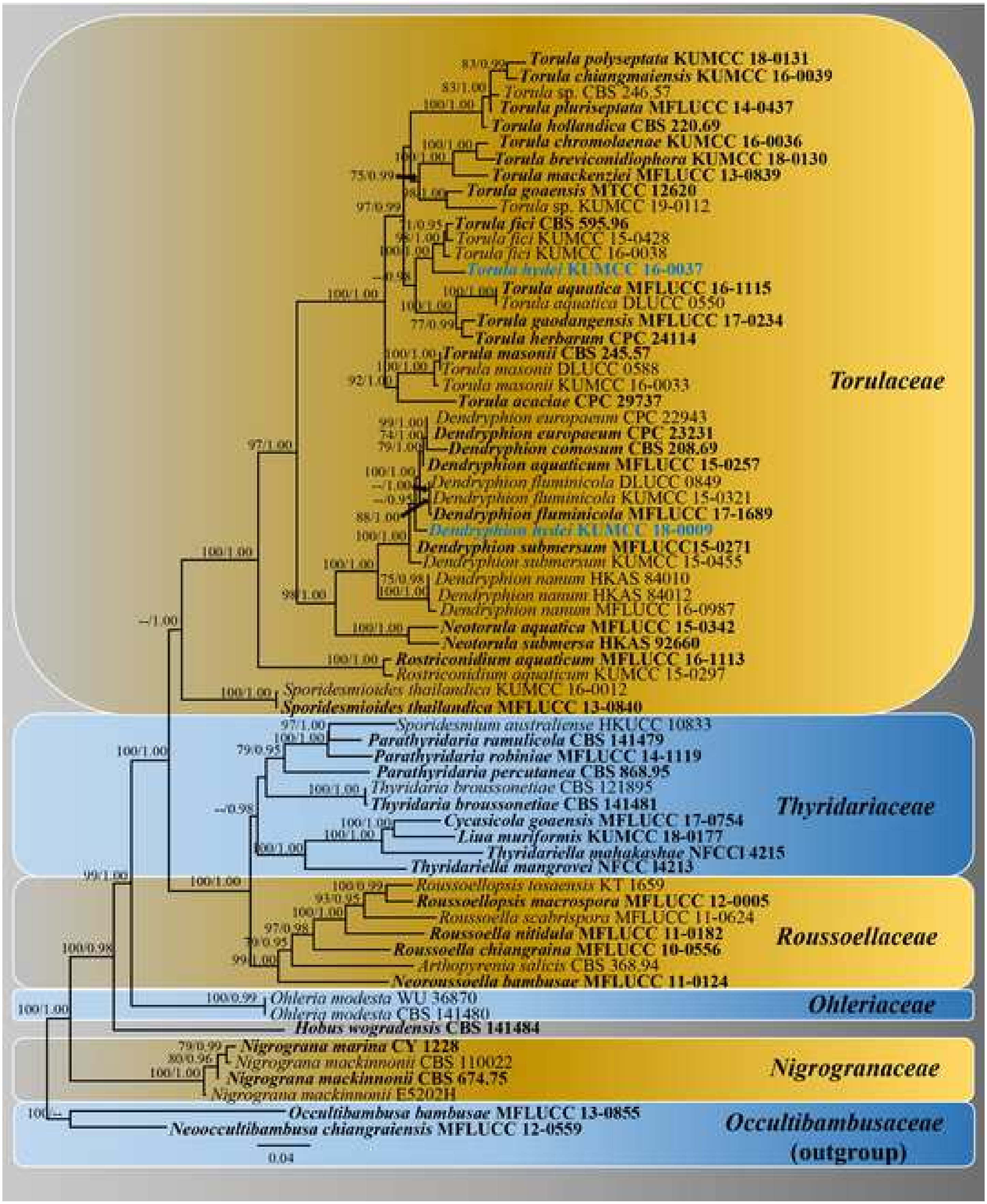

 Click here to download Figure Fig. 2 Dendryphion hydei.jpg

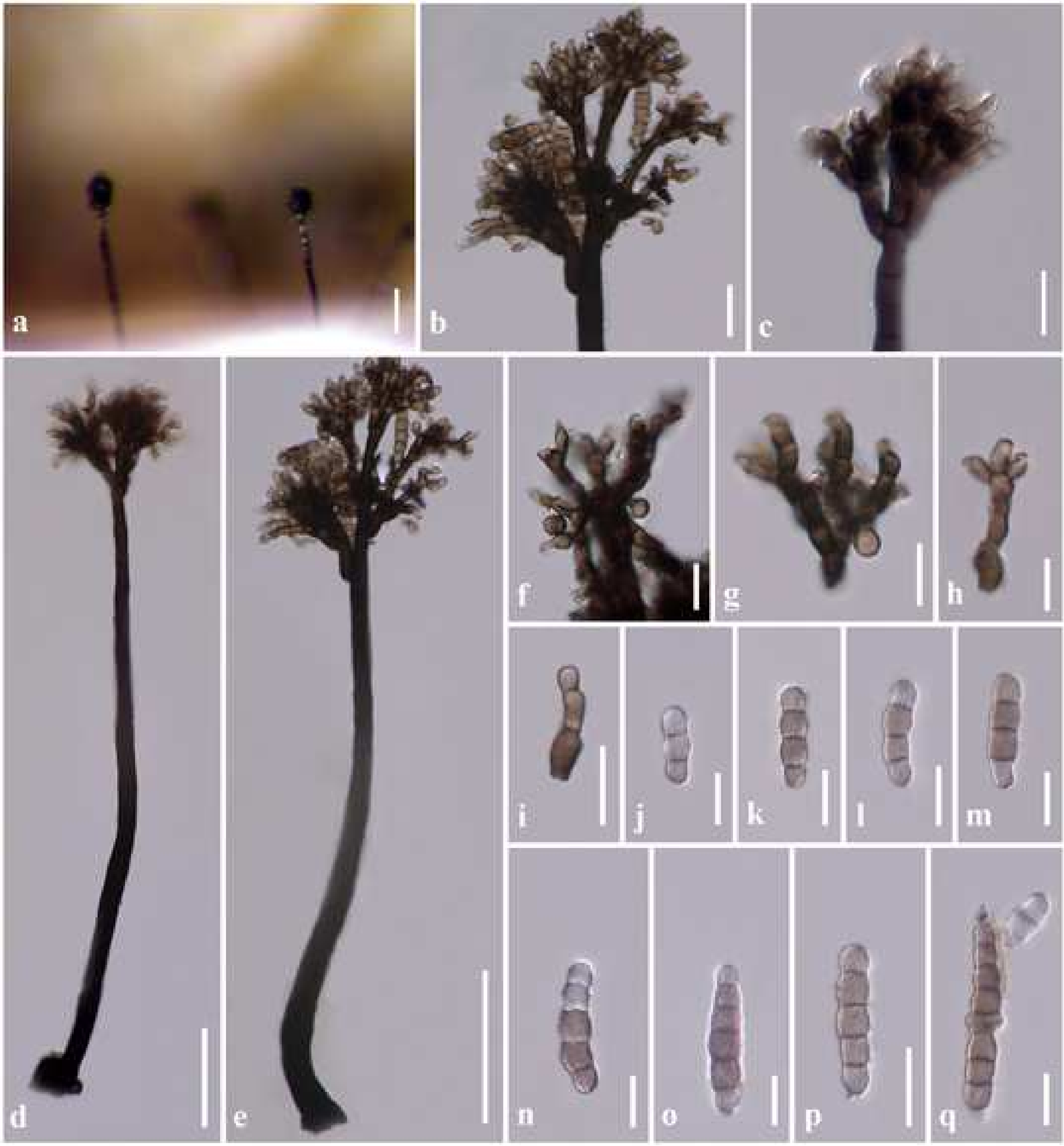

Click here to download Figure Fig. 3 Torula hydei .jpg

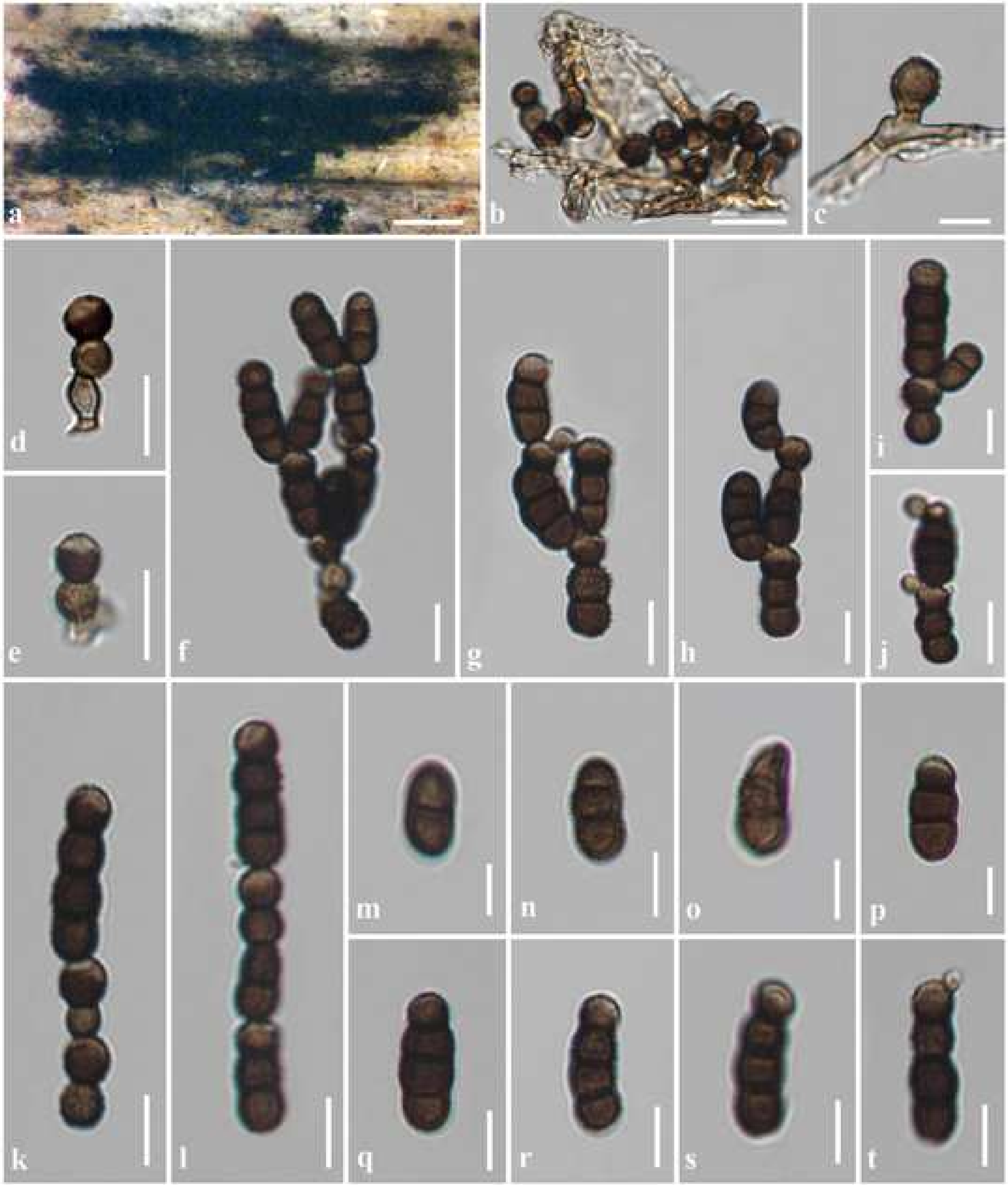

Supplement: Attachment — Submitted filename: PONE-D-19-27091_reviewer.pdf [file pone.0228067.s001.pdf]
