# Supplementary material for: Multigene phylogeny and taxonomy of Dendryphion hydei and Torula hydei spp. nov. from herbaceous litter in northern Thailand
Source: PLoS One. 2020 Feb 5;15(2):e0228067. doi: 10.1371/journal.pone.0228067 (PMC7001993; doi:10.1371/journal.pone.0228067)
Supplement: Attachment — Submitted filename: Response to Reviewers.docx [file pone.0228067.s002.docx]

**Dear Academic editor and reviewers,**

Thank you very much for your valuable comments and suggestion. We have revised our manuscripts following your comments and the revisions are highlighted in yellow in the revised manuscript. The journal format has checked and improved followed the PLOS ONE template. The GenBank accession and the Index Fungorum numbers were registered as well as the final alignment and multigene phylogenetic tree are also deposited in TreeBASE. We have removed the phrase “data not shown” from our manuscript and provide the low bootstrap support on our phylogenetic tree and manuscript. The detailed information for the response to reviewers are provide as below. If you prefer more information or any requirement for improving our manuscript. Please feel free to contact us directly. I will appreciate if our manuscript is considered to publish in PLOS ONE.

**Yours sincerely,**

Jun-Fu Li, Rungtiwa Phookamsak and Itthayakorn Promputtha

**Journal Requirements:**

1. When submitting your revision, we need you to address these additional requirements. Please ensure that your manuscript meets PLOS ONE's style requirements, including those for file naming.

**Response:** We have revised our manuscript following the template of PLOS ONE. However, we are not sure that we have to provide the Keywords for the manuscript or not. Therefore, the keywords are still available in our manuscript. Please let us know if this part needs to be removed. The title of the article is changed from “Multigene phylogeny and taxonomy of *Torula hydei* and *Dendryphion hydei* spp. nov. from herbaceous litter in northern Thailand” to Multigene phylogeny and taxonomy of *Dendryphion hydei* and *Torula hydei* spp. nov. from herbaceous litter in northern Thailand” following the alphabetical order of the taxon’s names.

2. Please take this opportunity to be sure you have met all of our guidelines for new species. When publishing papers that describe a new fungal taxon name, PLOS aims to comply with the requirements of the International Code of Nomenclature for algae, fungi, and plants (ICN). The following guidelines for publication in an online-only journal have been agreed such that any scientific fungal name published by us is considered effectively published under the rules of the Code. Please note that these guidelines differ from those for zoological nomenclature.

**Response:** We have followed the guidelines to describe the novel species and provide the nomenclature in the material and methods.

3. We note that you have stated that you will provide repository information for your data at acceptance. Should your manuscript be accepted for publication, we will hold it until you provide the relevant accession numbers or DOIs necessary to access your data. If you wish to make changes to your Data Availability statement, please describe these changes in your cover letter and we will update your Data Availability statement to reflect the information you provide.

**Response:** The GenBank accession and the Index Fungorum numbers were registered as well as the final alignment and multigene phylogenetic tree are also deposited in TreeBASE.

4. We note that you have included the phrase “data not shown” in your manuscript. Unfortunately, this does not meet our data sharing requirements. PLOS does not permit references to inaccessible data. We require that authors provide all relevant data within the paper, Supporting Information files, or in an acceptable, public repository. Please add a citation to support this phrase or upload the data that corresponds with these findings to a stable repository (such as Figshare or Dryad) and provide and URLs, DOIs, or accession numbers that may be used to access these data. Or, if the data are not a core part of the research being presented in your study, we ask that you remove the phrase that refers to these data.

**Response:** The phrase “data not shown” is removed from our manuscript and provide the low bootstrap support on our phylogenetic tree and the phylogenetic result.

5. Please remove any funding-related text from the manuscript and let us know how you would like to update your Funding Statement.

**Response:** The financial statements are removed from the acknowledgement and we would like to include our research funding to the funding statements as follows “The authors are grateful the Mushroom Research Foundation, Chiang Mai, Thailand and the Key Research Program of Frontier Sciences of the Chinese Academy of Sciences (grant no. QYZDY-SSW-SMC014) for supporting this research. Rungtiwa Phookamsak thanks CAS President’s International Fellowship Initiative (PIFI) for young staff (grant no. Y9215811Q1) and National Science Foundation of China (NSFC) project code 31850410489 (grant no. Y81I982211) for financial support. Peter E Mortimer would like to thank the National Science Foundation of China and the South East Asian Biodiversity resources Institute, Chinese Academy of Sciences, for financial support under the following grants: 41761144055, 41771063, Y4ZK111B01”.

**Reviewers' comments:**

**Reviewer #1:** Dear Junfu Li and co-authors,

Please see the attachment. I have included several comments in the attached manuscript. Basically, this is a well written manuscript, however, i am not sure the content is strong enough for the Plos ONE. Please remove the unnecessary paragraph from the introduction, and please improve your discussion. Most parts related to the Discussion should move to the notes part.

Please improve your phylogeny by including *Rutola.* Please refer "The Genera of Fungi – G5: *Arthrinium*, *Ceratosphaeria*, *Dimerosporiopsis*, *Hormodochis*, *Lecanostictopsis*, *Lembosina*, *Neomelanconium*, *Phragmotrichum*, *Pseudomelanconium*, *Rutola*, and *Trullula*"

**Response:** We have revised all changes following your comments and also improved the discussion. The phylogenetic analyses are updated by included *Rutola* and other missing strains in Torulaceae following Crous et al. (2020).

- **Keywords (L 40):** “2 new species” is replaced by new species.
- **Introduction (L 43):** “The family Torulaceae Corda was validly introduced …” is replaced by “The family Torulaceae Corda was introduced …”
- **Introduction (L 67–75):** “Based on the LSU rDNA sequence analysis, Crous et al. [8] reported two new species, *T. fici* Crous [as 'ficus'] and *T. hollandica* Crous. Li et al. [30] introduced four new species, *T. chiangmaiensis* Jun F. Li, Phook. & K.D. Hyde, *T. chromolaenae* Jun F. Li, Phook., Mapook & K.D. Hyde, *T. mackenziei* Jun F. Li, Phook. & K.D. Hyde and *T. pluriseptata* Jun F. Li, Phook., Camporesi & K.D. Hyde based on the analysis of a combined LSU, SSU, TEF1-α and RPB2 sequence dataset. Su et al. [48] introduced *T. aquatica* Z.L. Luo, K.D. Hyde, X.J. Su & H.Y. Su based on phylogenetic analyses of the combined ITS, LSU, RPB2 and TEF1-α sequence data. Hyde et al. [22] introduced *T. breviconidiophora* C.G. Lin & K.D. Hyde and *T. polyseptata* C.G. Lin & K.D. Hyde based on the analysis of the combined ITS, LSU, SSU and TEF1-α sequence data” is removed from the manuscript.
- **Introduction (L 86–90):** “Su et al. [47] circumscribed genera of Torulaceae from freshwater habitats and introduced two *Dendryphion* species, *D. aquaticum* Hong Y. Su & K.D. Hyde and D*. submersum* Hong Y. Su & K.D. Hyde and designated a reference specimen of *D. nanum* (Nees) S. Hughes based on molecular phylogeny. Su et al. [48] also introduced *D. fluminicola* Z.L. Luo, D.J. Bhat & K.D. Hyde.” is removed from the manuscript.
- **Isolation and identification (L 115):** “…malt extract agar (MEA) plates and grown at 16–30°C in …” is replaced by “…malt extract agar (MEA) plates and grown at room temperature (16–30°C) in …”. We grew the fungal culture at room temperature which is not specific control. it is around 16–30°C during the month that we isolated our fungi in the room of culture preservation.
- **Isolation and identification (L 119):** “Ex-type living cultures are deposited” is replaced by a. Ex-type living cultures were deposited.
- **Nomenclature (L 171–188):** “The electronic version of this article in Portable Document Format (PDF) in a work with an ISSN or ISBN will represent a published work according to the International Code of Nomenclature for algae, fungi, and plants, and hence the new names contained in the electronic publication of a PLOS ONE article are effectively published under that Code from the electronic edition alone, so there is no longer any need to provide printed copies.

In addition, new names contained in this work have been submitted to Index Fungorum from where they will be made available to the Global Names Index. The unique Index Fungorum number can be resolved and the associated information viewed through any standard web browser by appending the Index Fungorum number contained in this publication to the prefix www.indexfungorum.org/. The online version of this work is archived and available from the following digital repositories: PubMed Central and LOCKSS.”. The nomenclature is provided and revised based on the journal requirement.

- **Table 2 (L 275):** *Dendryphion hydei* has changed the position to the top.
- **Notes of *Torula hydei* (L 303)**: We revised the note of *Torula hydei* by providing more detail and a comparison of ITS and TEF1-α gene regions as follows “*Torula hydei* resembles *T. herbarum* and *T. fici* in having 2–3-septate, catenated, brown, verruculose conidia, but differs in having smaller conidia [10]. Phylogenetic analyses showed that *T. hydei* constitutes an independent lineage basal to *T. fici* (100% ML and 1.00 BYPP). Morphologically *T. hydei* differs from *T. fici* in having smaller conidia (*T. hydei*, (7.5–)8–14 × 2–4 µm versus (12–)13–17(–19) × 5(–6) µm, *T. fici*) and the conidia are also brown to dark brown, paler at the apex where branching occurs [8]. Whereas, *T. fici* has brown conidia, with a pale brown apex and the fertile cells in the conidial chain, where branching occurs, are darker brown than other cells [8]. The conidiogenous cells of *T. fici* are slightly larger than *T. hydei* and frequently clavate (*T. fici*, (5–)6(–8) × 5(–7) µm versus 3–5.5 × 4.3–5 µm, *T. hydei*), whereas, *T. hydei* has doliiform to ellipsoid conidiogenous cells [8]. We also note distinct nucleotide base pair differences between *T. hydei* and *T. fici* (CBS 595.96, type strain) across the ITS gene region (8/479 bp, 1.7% difference, no gap) and TEF1-α gene region analysed (43/760 bp, 5.7% difference, no gap). Based on distinct morphological characteristics and phylogenetic support, *T. hydei* is introduced as a new species in this study”.
- **Discussion (L 327–341):** We have already moved “Our new taxon, *Torula hydei* is characterized by morphs that correspond to those outlined by Li et al. [30]. However, *T. hydei* is unique in having very tiny conidia as compared to other similar species. We also note distinct nucleotide base pair differences between *T. hydei* and *T. fici* across TEF1- α gene region analysed (43/760 bp, 5.7% difference)” to the notes of *T. hydei* and also compare the nucleotide base of ITS region. “*Dendryphion hydei* is unique in having large conidiophores and subhyaline to pale olivaceous brown, 2-4(-5)-septate conidia to compare with other related species in *Dendryphion* (Table 2). Our multiloci phylogeny also positions our new taxon as independent lineage and phylogenetically apart from other species (Fig. 1). A comparison of TEF1-α nucleotides shows that *Dendryphion hydei* differs from *D. fluminicola* in 20/852 bp (2.3% difference), from *D. submersum* in 30/902 bp (3.3% difference). A comparison of ITS nucleotides shows that *D. hydei* differs from *D. europaeum* in 19/553 bp (3.4% difference) and differs from *D. aquaticum* in 6/398 bp (1.5% difference). Phylogenetic analyses support *D. hydei* as a new species in *Dendryphion*. These tally with recommendations outlined by Jeewon and Hyde [26] to establish our new species” is also moved to the notes of *Dendryphion hydei*.
- **Table 3 (L 349):** The information of the genus *Rutola* is included to the table 3.

**Reviewer #2**: Note: The following minor corrections may please be attended -

Page 8, Lines 26-27: Abstract: Delete the first sentence. Start the paragraph from second sentence – During our studies....

Pages 8-9, Lines: 44-48: Replace the sentences with this: Species in Torulaceae are known only by their asexual morphs which are characterized as follows: Superficial, effuse, greyish brown to black, powdery colonies; micro- or macronematous conidiophores, with or without apical branches; doliiform to ellipsoid or clavate, brown, smooth to verruculose, mono- to polyblastic conidiogenous cells which often remaining cupulate; subcylindrical, phragmosporous, acrogenous, brown, dry, smooth to verrucose conidia characteristically produced in branched chains

Page 9, L.50: accepted *Dendryphio*n Wallr., besides *Torula,* within Torulaceae in Pleosporales.

Page 9, L.54: relationships of the taxa within the family ...

Page 11, L.94: northern Thailand. Among collected samples, *Dendryphion hydei* is recovered as another new .....

Page 11, L.96: updated phylogenetic tree with our new taxa for the family *Torulaceae* is provided in this ...

Page 12, L.113: Single conidium isolation ..

Page 24, L. 287: semi-macronematous, mononematous, ....

Page 24, L. 289: subglobose, arising from prostrate hyphae

Page 25, L. 315: al. [8] who re-classified Torula and ....

Page 25, L. 316: ......... Subsequent authors introduced new genera ....

Page 25, L. 320: Fungorum [23], but most of the described species lack DNA .....

**Response:** We have done all changes following the reviewer2’s commented and the revisions are highlighted in yellow in the “Revised Manuscript with Track Changes” file.
